# Investigation into the Reinforcement Modification of Natural Plant Fibers and the Sustainable Development of Thermoplastic Natural Plant Fiber Composites

**DOI:** 10.3390/polym16243568

**Published:** 2024-12-20

**Authors:** Zhenhao Liao, Yiyun Hu, Yan Shen, Ke Chen, Cheng Qiu, Jinglei Yang, Lei Yang

**Affiliations:** 1 College of Civil and Transportation Engineering, Shenzhen University, Shenzhen 518060, China; liaozhenhao2020@email.szu.edu.cn (Z.L.); huyiyun2023@email.szu.edu.cn (Y.H.); chenke23@email.szu.edu.cn (K.C.); 2 Department of Mechanical and Aerospace Engineering, Hong Kong University of Science and Technology, Hong Kong 999077, China; yshenbl@connect.ust.hk (Y.S.); maeyang@ust.hk (J.Y.); 3 Institute of Mechanics, Chinese Academy of Sciences, Beijing 100190, China; qiucheng@imech.ac.cn; 4 HKUST Shenzhen-Hong Kong Collaborative Innovation Research Institute, Shenzhen 518031, China

**Keywords:** natural plant fibers, physical treatment, chemical treatment, thermoplastic resin, FRP

## Abstract

Natural plant fibers (NPFs) have emerged as a sustainable alternative in the manufacture of composites due to their renewability and low environmental impact. This has led to a significant increase in the use of natural plant fiber-reinforced polymers (NPFRPs) in a variety of industries. The diversity of NPF types brings a wide range of properties and functionalities to NPFRPs, which in turn highlights the urgent need to improve the properties of fiber materials in order to enhance their performance and suitability. This paper provides insight into the processing mechanisms behind NPF fiber treatments, exploring how these treatments affect the mechanical, thermal and environmental properties of NPFRPs. It also offers a critical assessment of the advantages and disadvantages of physical, chemical, biological and nanotechnological treatments. The findings of our analysis provide a basis for the development of future treatments that aim to enhance the material properties of NPFRPs, thereby increasing their competitiveness with conventional synthetic fiber-reinforced polymers. Finally, a novel thermoplastic resin composite system, Elium–NPFRP, is proposed that embodies the principles of green development. The system has been designed with the objective of capitalizing on the environmental benefits of NPFs while simultaneously addressing the challenges associated with the integration of NPFs into polymer matrices. The Elium–NPFRP composite system not only exemplifies the potential of NPFs for sustainable materials science, but is also a practical solution that can be implemented in a diverse range of applications, spanning automotive components to construction materials. This has the potential to reduce carbon footprints and promote a circular economy.

## 1. Introduction

In the global arena, governments of various nations and international organizations are committed to fostering a low-carbon economy and sustainable development as a response to the challenges posed by climate change and environmental degradation. Against this backdrop, natural plant fiber-reinforced polymers (NPFRPs) have emerged as a pivotal direction in the field of materials science due to their exceptional environmental attributes and sustainability [1].

NPFRPs are composite materials that integrate renewable plant fibers such as flax, jute, bamboo, and sisal with polymers [2]. These composites not only diminish reliance on fossil fuels but also exhibit a lower carbon footprint throughout their lifecycle. The utilization of such materials aligns with the low-carbon policies of multiple countries and regions, such as the European Union’s Green Deal, the United States’ BioPreferred Program, and China’s Circular Economy Promotion Law. These policies encourage the adoption of circular economy principles, aim to reduce greenhouse gas emissions, and promote the research and application of sustainable materials.

**Figure 1:**
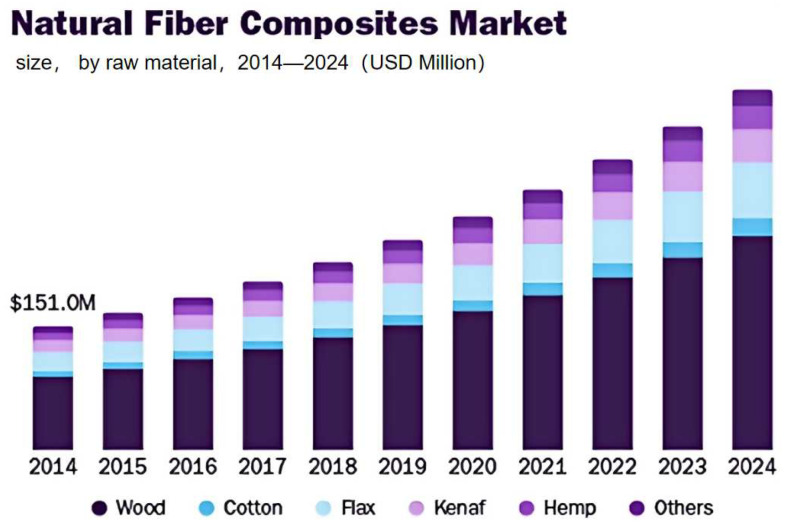
Natural fiber composites market between 2014 and 2024 (retrieved from Grand View Research).

NPFRPs are emerging as key materials in driving sustainable development across various industries. From lightweight components in the automotive sector to eco-friendly construction materials and ecologically benign casings for electronic devices, these materials meet the dual demands of modern society for environmental protection and efficiency, thanks to their renewability, low life-cycle carbon footprint, and superior performance [3]. For instance, in June 2024, Bcomp introduced the world’s first car (Quintessenza™) to incorporate linen fiber composite materials into multiple external components, including the front and rear bumpers, side skirts and wheel arches, showcasing an innovative integration of materials. Compared with carbon fiber composites, the carbon dioxide emissions were reduced by 85%. Prior to this, Bcomp collaborated with Tesla to launch a racing car (EPCS V2.3 Tesla P100DL), which utilized natural plant fiber (NPF) composites for the car body, resulting in a weight reduction of 500 kg compared with the alloy version. Currently, numerous industries are exploring the vast application potential of NPFRPs [4]. By 2024, the global market size for natural fiber composites is projected to reach USD 10.89 billion, with an anticipated compound annual growth rate of 11.8% during the forecast period. Figure 1 shows the natural fiber composites market from 2014 to 2024.

NPFRPs, despite demonstrating their environmental and sustainability advantages in various fields, still possess some inherent drawbacks that limit their widespread application. These shortcomings include relatively low mechanical strength, insufficient durability, and sensitivity to humidity and environmental changes. For instance, the hygroscopic nature of NPFs can lead to a degradation of material performance over time, particularly in humid environments. Moreover, the bonding performance between NPFs and certain polymers may not be optimal, affecting the overall performance of the composite materials [5].

To overcome these limitations and maximize the potential of NPFRPs, fiber surface modification treatments are particularly important. Therefore, research on the surface enhancement of NPFs is essential for the development of high-performance NPFRPs. This involves techniques such as chemical treatments, plasma treatments, or the application of coupling agents to improve the interfacial adhesion between the fibers and the matrix, thereby enhancing the mechanical properties and durability of the composites [6].

Additionally, the material performance of NPFRPs is not only affected by NPFs, but also by the reasonable selection of polymers. The thermoplastic resin can be recycled through certain processes due to its heat–melt–reformable characteristics, which can effectively reduce the carbon footprint of the entire composite material, and the combination of NPFRPs with NPFs will offer better sustainability than traditional composites (e.g., CFRP, GFRP, etc.). Thermoplastic NPFRPs not only provide mechanical performance enhancement, but also support circular economy, help reduce greenhouse gas emissions, and promote environmental innovation. The development of new resins that are more compatible with NPFs and the exploration of novel manufacturing processes can further address these challenges and expand the range of applications for NPFRPs. With technological progress, the innovative application prospects of this type of composite materials are infinitely broad. Section 4 of this article provides a comprehensive account of the evolution of NPFRPs, the utilization of diverse resin systems in conjunction with NPFs, and the multifaceted benefits of NPFRPs. In addition to an evaluation of surface treatment techniques for NPFs, which are pivotal for enhancing the interfacial bond strength of NPFRPs, the article also investigates the prospective applications of NPFRPs in lightweight structures, biodegradable products, and environmentally conscious endeavors.

In the current study, the key challenge is to enhance the performance of NPFs in NPFRPs by improving their surface treatment techniques and to explore the potential of these composites in sustainable development. Specifically, this research aims to address the lack of mechanical properties, durability limitations, and sensitivity to environmental changes of NPFs. It will also explore the application of thermoplastic resins in NPFRPs, particularly the Elium resin system, with a view to achieving more environmentally friendly and recyclable composite solutions. Through the comprehensive analysis of these research issues, we expect to provide a scientific basis for the future development of NPFRPs and promote their application in multiple industries.

## 2. The Characteristics of Natural Plant Fibers

### 2.1. Types of NPF

Natural fibers originate from plants, animals, and minerals [7]. Most plant fibers are mainly composed of cellulose, while protein constitutes the main component of animal fibers. There are three main types of animal fibers [8], animal hair (wool, etc.), poultry fibers, and silk fibers, although animal fibers have great potential for utilization, the production of animal fibers may involve the use of animals, which may in some cases lead to controversies in animal welfare and ethics. Common natural mineral fibers include [9] silicate mineral fibers, the vast majority of which may contain harmful substances or produce harmful by-products during processing, thus necessitating caution in their use.

There are six main types of common NPF [10]: Bast fibers (such as jute, ramie, flax, hemp, apocynum, hibiscus and kenaf), which are collected from the epidermis of plants; leaf fiber (banana, sisal, and pineapple); seed fibers (cotton, coconut shell, and kapok); stem dry fiber (corn and wheat straw); root fiber (luffa and cassava); and fruit fiber (tamarind).

**Figure 2:**
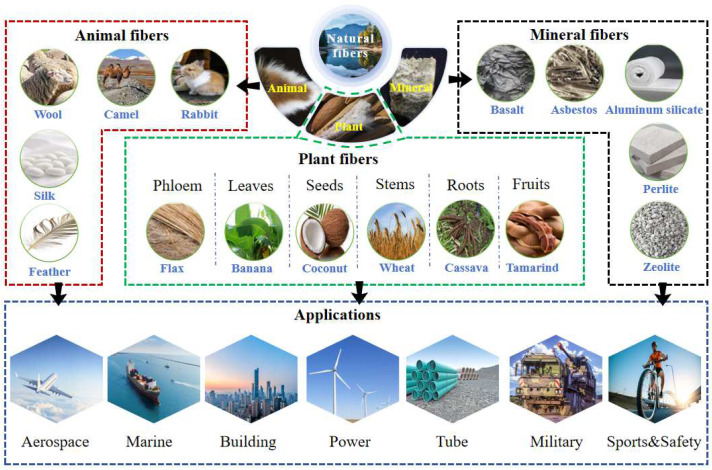
Types and applications of natural fibers (images sourced via the internet).

Compared with animal and mineral fibers, NPFs have advantages such as renewability, fewer ethical issues, and environmental friendliness, making them an ideal choice in sustainable development strategies. Figure 2 shows the types and applications of natural fiber. In the vast majority of literature, natural fibers refer to NPFs. The following discussion in this article focuses only on NPFs.

With the advancement and innovation of genetic engineering technology, plant varieties with stronger fiber strength and durability can be cultivated by improving the genetic characteristics of plants, demonstrated when Liu [11] achieved a 118% increase in fiber length by controlling favorable alleles on cotton fibers in a breeding line with a fiber length of 33.8 mm. Abdel Aty [12] improved Egyptian cotton through genetic hybridization technology, and their results were found to show that hybrid cotton varieties had higher seed cotton yield, lint cotton yield, and fiber quality, with fiber length values exceeding 31 mm.

At the same time, with the development of cellulose extraction technology, many new types of NPF can be extracted and developed. For example, Mulyani et al. [13] identified a new type of cellulose fiber from the Canna Edulis plant and extracted it through a water immersion process. The extracted fiber length is about 30 cm, and the average diameter of the fiber is between 50 and 60 μm. Vinod et al. [14] discovered a new type of cellulose fiber from Cathantanthus roseus. This was achieved by collecting, cleaning, and soaking the plant stems in water for 3 days for fiber separation, followed by a mechanical soaking process. The extracted fibers were treated with 5% alkali, with a length of 5–10 cm, a diameter of 290.2 μm, and a density of 1.34 g/cm^3^. Additionally, new fibers, such as centaurea solsticalis, cereus hildmannianus, chrysanthemum morifolium, cissus quadrangularis, and cissus vitiginea, have good material properties and great development and application prospects [15].

With the improvement of NPF types and properties, the application prospect of NPFRPs is becoming ever broader. In the future, NPFRPs will play an important role in civil engineering, building structures, wind energy, and automotive and railway transport due to its light weight, high strength and environmentally friendly properties. Especially in precast component manufacturing, NPFRP’s ease of processing makes it an ideal material choice. In addition, NPFRPs are increasingly used in thermoplastic matrix composites, thanks to their fast processing and recyclability. The development of liquid molding technology and composite recycling technology will further contribute to the development of the multifunctionality of NPFRPs, such as flame retardant, antistatic and antimicrobial properties, to meet the needs of specific industries. These applications of NPFRPs not only contribute to the advancement of materials science, but also provide a strong support for the achievement of sustainable development strategies.

### 2.2. The Main Components of NPF

**Figure 3:**
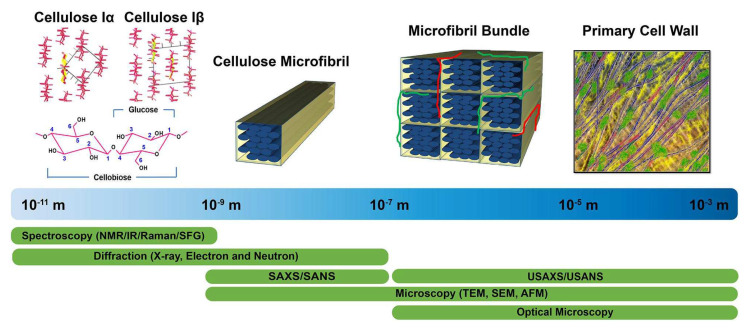
Diagram of cellulose composition in an NPF [21].

The primary constituent of most NPFs is cellulose, a complex carbohydrate composed of glucose molecules linked by β-1,4-glycosidic bonds [16], (as shown in Figure 3). It is evident that the chemical and physical properties of cellulose are the main determinants of the material properties of NPFs [17]. In industrial applications, cellulose with varying degrees of crystallinity and unit cell sizes can influence the processing methods and the performance of the final product. For instance, the high crystallinity of cellulose and the strength of intermolecular hydrogen bonds confer significant mechanical strength on NPFs [18]. However, NPFs also contain other components, such as hemicellulose and pectin, whose presence affects the softness and plasticity of the fibers [19]. Consequently, some scholars [20] believe that adjusting the ratio of cellulose, hemicellulose, and pectin within the fibers through fiber treatment methods can help NPFs exhibit better material properties.

Therefore, before surface treatment of NPFs, it is essential to understand how the treatment methods will affect the physical and chemical properties of the fibers. In particular, comprehending the impact of chemical treatment methods on the cellulose within an NPF is crucial. This understanding will help us profoundly grasp the mechanisms of NPF modification and guide the design of future optimization plans.

### 2.3. Material Characteristics of NPF

The material properties of common NPFs depend mainly on their composition, microfiber angle (MFA), crystallinity, and internal structure [22]. Table 1 presents the material properties of common NPFs. The distinct composition and structure of the NPF largely dictate the characteristics of the relevant NPFRPs. For instance, the hardness of an NPF is particularly contingent upon MFA. NFPs with high cellulose content and low MFA typically exhibit greater fiber hardness as a fiber, as the low MFA aligns the fibers almost parallel to the direction of the tensile load, thereby enabling the fibers to withstand higher loads and in turn enhancing hardness.

It is worth noting that the superior strength and durability of flax fibers are primarily attributed to their lower MFA (2–8°) and higher cellulose content (30–76 wt%) [23]. NPFs with relatively high cellulose content also include cotton linters, cotton and ramie, among others. Essentially, fibers with high cellulose content and low lignin content exhibit better tensile strength; however, this correlation is not always linear due to the many factors that affect tensile strength. The degree of cellulose crystallinity has a significant impact on the tensile strength value; the higher the crystallinity of cellulose, the greater the strength of the fiber. The location of lignin in the biomass also affects tensile strength, causing a reduction in tensile strength, as lignin is interposed between cellulose and hemicellulose, affecting their interaction, thereby influencing the tensile strength of the NPF [24]. Therefore, a lower lignin content can enhance the purity of cellulose, which in turn enhances the fiber’s mechanical properties, including strength and stiffness.

**Table 1:** Material properties of NPFs [22,25,26,27,28,29,30,31,32,33,34,35,36,37,38,39,40,41,42,43].

Fiber		Chemical Composition						Physical Properties				
	Cellulose [%]	Hemi-Cellulose [%]	Pectin [%]	Micro-Fibrillar Angle [°]	Lignin [%]	Wax [%]	Ash [%]	Diameter (μm)	Density (g/m³)	Tensile Strength (MPa)	Tensile Modulus (GPa)	Elongation at Failure %
Abaca	56–63	15–17	0.5–1.8	20–25	7–9	3	3	10–30	1.5	430–813	31.1–33.6	2.9–10
Alfa	45	24	14.9		24	5			1.4	247	8.5	1.96
Bamboo	26–43	30			5–31	10		88–125	0.91–1.26	503	35.91	1.4
Bagasse	25–45	38–32			15–25				1.2	20–290	19–27	1.1
Banana	63–83	17–21	3–5	11–12	5	11		100–250	1.35	529–914	27–32	2.6–5.9
Coconut coir	32–43.8	0.15–20	3–4		40–45			1.15	100–450	500	2.5	3.36
Coir	36–43	41–45	3–4	30.45	0.15–0.25			150–250	1.15–1.25	131–220	4–6	15–40
Cotton	83–91	3–5.7	0.6			0.6			1.51	400	12	0.3–10
Curaua	73.6	9.9			7.5			7–10	1.4	87–1150	11.8–96	1.3–4.9
Flax	64–72	1.8–2	1.8–2.3	5–10	2–2.2	1.7		25	1.4	800–150	60–80	1.2–1.6
Hardwood	43–47				25–35				0.3–0.88	51–121	5.2–15.6	
Hemp	70–74	0.9	0.8	2–6.2	3.7–5.7	1.2–6.2	0.8	25–600	1.48	550–900	70	1.6–4.0
Henequen	75–78	4–8			13–14		3		1.2–1.4	430–570	10–16.3	3.7–5.9
Jute	61–72	18–22	0.2	8	12–13	0.5	0.5–2	25–250	1.3–1.48	393–800	0.13–26.5	1.16–1.8
Kenaf	45–57	8–13	0.6	2–6.2	22	0.8	2–5		1.25–1.40	284–930	21–60	1.6
Nettle	86	5.4	0.6		4	3.1		25~4	1.594	650	38	1.7
Oil palm	56–65	27.5			20.48		2.4		0.7–1.5	150–500	80–248	17–25
Palymyrah	58.58	22.8			13.48	0.35			1.09–1.38	276–281	8.9–22.9	
Pineapple	70–75	4–6			8–11	1–2	1–3	50	1.44	413–1627	60–82	14.5
Ramie	69–91	5–15	1.9		0.4–0.7			20–280	1.3–1.5	400–938	61.4–128	3.6–3.8
Rice husk	38–45						20		0.5–0.7			
Sea grass	57	5	38					1.5	5	453–692	3.1–3.7	13–26.3
Sisal	78	10			8	2		50–200	1.3–1.4	390–450	12–41	2.3–2.5
Softwood	40–44				25–29				0.30–0.59	45.5–111	3.6–14.3	4.4

Pectin and hemicellulose in NPFs have a significant impact on the water absorption, wet strength, and swelling properties of fibers [44]. Hemicellulose and pectin contain a large number of hydrophilic groups, such as hydroxyl (-OH), which can form hydrogen bonds with water molecules, thereby increasing the water absorption of fibers. The hydrophobicity and hydrophilicity of fibers, as well as their interaction with the matrix, can further affect the adhesion between fibers and resins [45]. Therefore, for composite applications, high pectin and hemicellulose content in the NPF makes the composites less dimensionally stable, and water absorption tends to lead to matrix cracking and reduced mechanical properties.

In addition, lignin molecules contain conjugated double bonds, which can absorb ultraviolet (UV) radiation and protect plants from UV radiation damage. Increasing the lignin content in materials can improve their ability to absorb UV radiation and reduce the direct damage of ultraviolet radiation to the NPF [46]. Kaya [47] and Valchev [48] both believe that an appropriate amount of lignin is beneficial for improving the corrosion resistance of an NPF. Therefore, exploring an appropriate amount of lignin in the future is an effective way to improve the corrosion resistance of NPFs, which is conducive to promoting their widespread application.

NPFs have a wide range of fiber diameters, fiber bundle widths and fiber lengths, leading to significant changes in the performance of natural fiber composites. In addition, these changes in the characteristics of an NPF pose significant challenges in optimizing the manufacturing process of using fibers as reinforcing materials. Most fibers require secondary processing before they can be used, but some fibers are too thin and short, and can only act on certain components with low requirements for fiber length. Fiber length limits the structural form of the components.

### 2.4. Processing Methods of NPF

The processing methods of different NPFs vary. The general process of processing an NPF (such as seed fibers) from raw materials into fiber bundles typically includes the following key steps.

- Preparation of raw materials: Raw materials for making nonwovens usually include fibers, adhesives and other auxiliary materials.
- Fiber blending: In the production of nonwovens, different types of fibers are often blended together to obtain the desired properties and characteristics. For example, blending fibers with different lengths can increase the strength and abrasion resistance of a nonwoven. The blending process is usually carried out by feeding the fibers into a mixer and mixing them homogeneously using mechanical agitation.
- Fiber pretreatment: Before fibers can be converted into nonwovens, a number of pretreatment operations need to be carried out on them. These operations include washing, dyeing, coating and drying. The cleaning process removes impurities and stains from the fibers to ensure fiber quality. Dyeing and coating give the fibers specific color and performance properties. The drying process removes moisture from the fibers to make them suitable for the next step in the process.
- Fiber bonding: Fiber bonding is a key step in the production of nonwovens. There are a number of ways to achieve fiber bonding, including needling, heat fusion, bonding and hydraulic autoclaving. In the needling method, fibers are interwoven with each other by using sharp needles to drive the fibers into the substrate at the bottom. The thermal fusion method fuses surrounding fibers to each other by thermally fusing their surfaces. The bonding method uses an adhesive to bind the fibers together and can be achieved by spraying, printing, or coating. The hydraulic high-pressure method uses a high-pressure stream of water to bind the fibers together.
- The final stage of the product processing is complete. Once the nonwoven has undergone fiber bonding, it is then subject to a finishing process. The finishing processes that may be applied to the product include coating, lamination, winding and cutting. Coating can be employed to impart specific properties to the nonwoven, including waterproofing, fireproofing, or anti-static properties. The lamination process serves to bond the nonwoven to other materials, thereby enhancing its functionality. Winding is a process whereby wide nonwovens are wound into rolls for subsequent storage and transport. Cutting reduces the dimensions of a nonwoven material to the requisite size and shape.
- Wet lay-up is an emerging technology for the production of nonwovens utilizing natural cellulose staple fibers and their blends. The process comprises three principal steps: dispersion, deposition and consolidation. The attainment of defect-free nonwovens during the web formation process hinges upon the achievement of uniform dispersion. The methodology bears resemblance to the papermaking process, although the fiber length and fiber density are distinct throughout the process. The steps outlined can be adapted according to the type of natural fiber and the specific requirements of the end product. Amutha [49] employed a manual stripping process coupled with a retting process using water and enzymatic treatments to extract banana fibers from the banana silk. The extracted fibers had a high cellulose content of 56.48% and a low wax content of 1.05%. Additionally, the fibers measured approximately 25 cm in length, with tensile strength and Young’s modulus of 178 MPa and 3.0 GPa, respectively.

In recent years, several innovative techniques, such as ultrasonication [50], microwave irradiation [51,52], and low-temperature treatment [53], have been applied to the extraction of NPFs [54]. Li [55] proposed a novel method that combines microwave and ultrasonication for fiber extraction. Compared with the traditional chemical degumming process, this new approach has reduced the degumming time and reagent consumption by 31.2% and 44.8%, respectively, while yielding high-quality hemp fibers. Microwave technology holds promising prospects for the extraction of NPFs, and in the future, it could be integrated with advanced techniques such as low-temperature processing to optimize the material properties of NPFs, thereby enhancing their performance and applicability in industrial applications.

## 3. Surface Treatment Methods for Natural Plant Fibers

The differences in structure and composition of NPFs determine the selection of their surface treatment methods, the common processing methods include physical and chemical methods.

### 3.1. Physical Method

In order to understand the impact of physical processing on NPFs, extensive research has been conducted. Important physical methods include steam explosion, high-pressure sterilization, corona charging, and high-energy radiation.

#### 3.1.1. Steam Explosion Treatment

**Figure 4:**
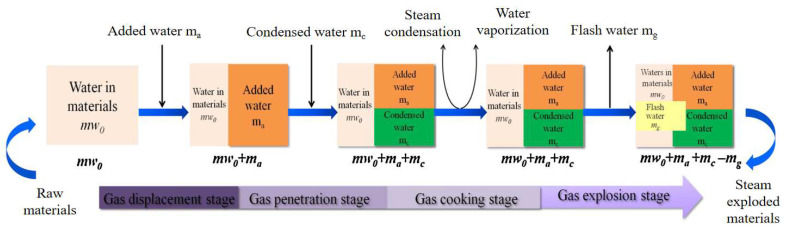
The steam explosion process [56].

Steam explosion (SE), also known as self-hydrolysis, is an atypical method of hydrothermal pretreatment [56]. According to the SE principle, the raw material is placed in a cylinder at high temperature and pressure, and the steam generated is used to penetrate the interior of the material, filling the pores of the tissue with steam. Finally, the high pressure from the saturated steam is immediately released within milliseconds. The water contained in the fibers evaporates and expands rapidly, causing the cell walls to rupture and form pores, thus releasing small molecules from the plant cells. This also leads to reduced cellulose crystallinity, to delignification, and hemicellulose hydrolysis [57,58]. The process is widely used in the pretreatment of lignocellulosic biomass as it is an effective and environmentally friendly treatment without the addition of chemicals. Figure 4 demonstrates the SE processing. Pérez-Limiñana et al. [59] used the SE method to treat palm leaf fibers and showed effective removal of lignin and hemicellulose and a 30% increase in the crystallinity of α-cellulose. Lorenzo-Hernando [60] investigated the treatment of coniferous fibers with SE at 150 °C and 200 °C, respectively, and showed that the crystallinity of the fibers decreased after treatment at 150 °C, whereas recrystallisation of the cellulose took place at 200 °C. Nattaporn [61] treated hemp fibers with SE and combined it with polylactic acid resins to form NPFRPs, and it was found that the flexural modulus of the SE-treated NPFRPs was enhanced by 105%.

#### 3.1.2. Plasma Treatment

**Figure 5:**
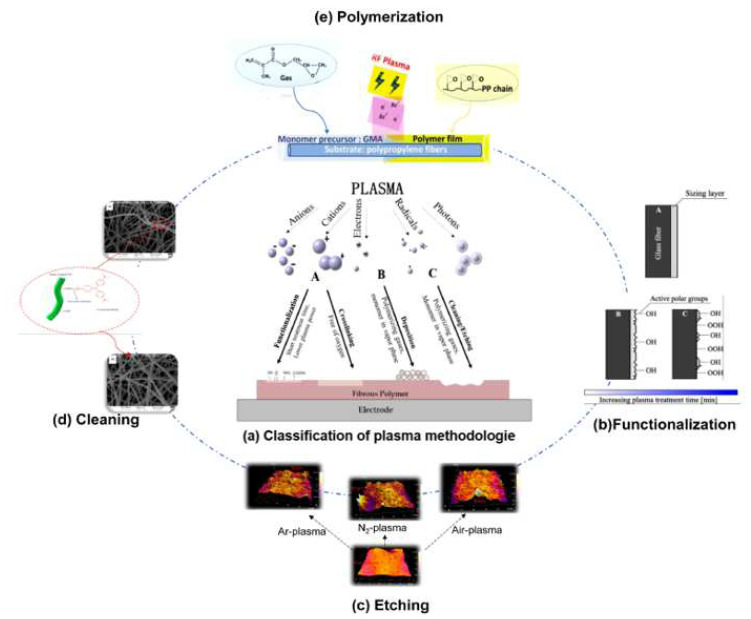
Surface etching and functionalization of fiber surfaces by plasma treatment [66].

Plasma treatment generally requires the fibers to be in a vacuum chamber where gases are then introduced and ionized to produce plasma. The gases used include oxygen, nitrogen, helium, and air. Ions of the introduced gases strike the fiber surface, causing physical and chemical changes in the fiber’s structure [62]. Figure 5 illustrates the process of the plasma treatment of fibers. Gassan and Gutowski [63] have demonstrated that plasma treatment can change the surface energy of cellulose and thus improve the compatibility between an NPF and resin. Olivier [64] used fluorine gas as plasma to treat flax fibers and showed that the tensile modulus of the flax fibers was reduced by 2% and that the polarity of the fibers was reduced. Mohsen [65] used atmospheric plasma to treat flax fibers and the treated flax fibers were formed into an NPFRP with a polybutyl succinate resin. Additionally, it was found that the flexural strength of the NPFRPs was increased by 18% and that the impact strength was improved by 10%.

#### 3.1.3. Radiation Treatment

**Figure 6:**
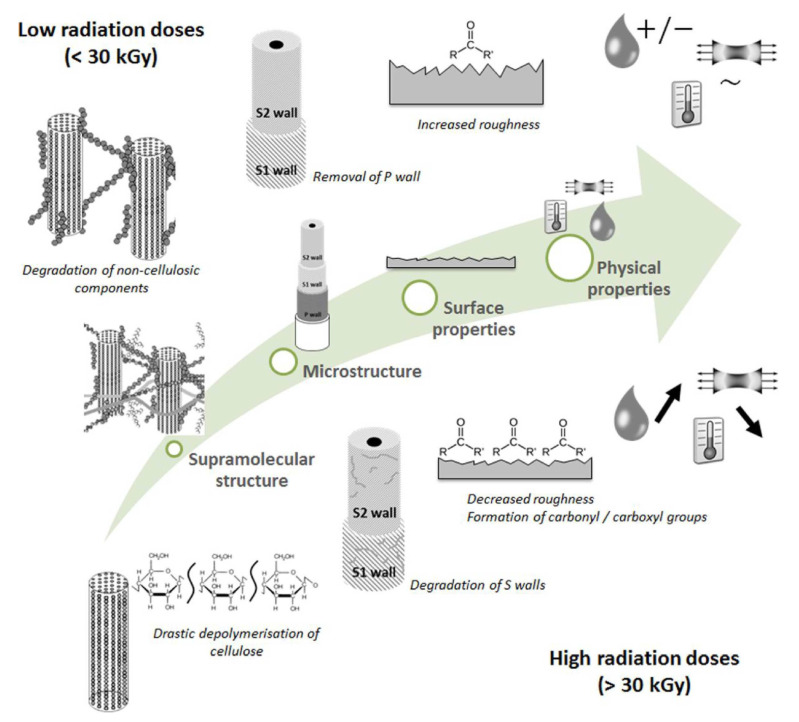
Summary of the main chemical and structural changes occurring upon irradiation of an NPF from the supramolecular structure to the fiber surface, and the effect on their physical properties [73].

The main mechanisms of radiation affecting fibers involve changes in chain structure and molecular conformation, which are primarily manifested in changes in the degree of polymerization and chemical group transitions. When a certain threshold is reached, these changes cause changes in mechanical properties, including strength and color fastness [67]. Some of the common radiation treatments are UV and gamma radiation [68]. The mechanism of the effect of radiation on NPFs is illustrated in Figure 6. Ghanayem [69] treated cotton fibers with electron beam radiation, resulting in improved hygroscopic properties. Henniges [70] treated pulp with electron beam radiation, which resulted in a reduction in the relative crystallinity of microcrystalline cellulose from 87% to 45%, and the treated pulp was easier to color. In a study by Hidzir [71], rice husk ash was irradiated with gamma rays at a dose of 7 kGy and it was found that interfacial bonding within the materials was enhanced after irradiation. The radiation treatment of fibers, followed by a combination of other methods for enhancing fiber bonding properties, is a multi-step, or hybrid, method. Such a method can take advantage of the strengths of different treatments, such as radiation followed by grafting or plasma treatment followed by grafting [72], where the use of a combination of physical and chemical methods is already widespread, but there is a need to explore more treatments in the future to further enhance the material properties of fibers.

### 3.2. Chemical Treatment

**Figure 7:**
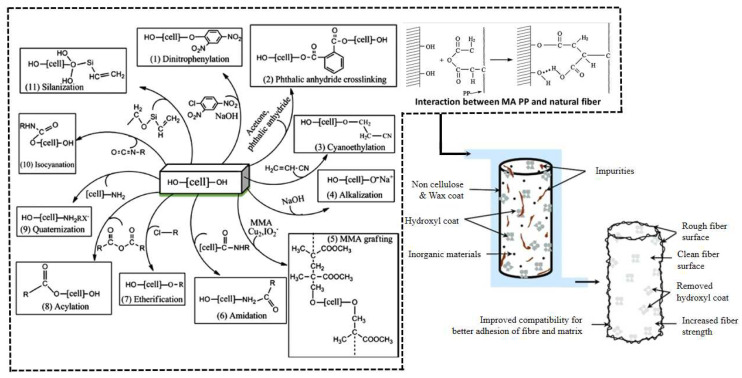
Summary of methods used to modify fibers’ surface [77,78].

NPFs are usually highly hydrophilic due to the presence of a large number of polar groups, such as hydroxyl groups, in their molecular structure. These groups readily form hydrogen bonds with water molecules, resulting in fibers with good water absorption. However, this hydrophilicity results in poor compatibility between NPFs and non-polar resin matrices, thus limiting their use in composites. Common chemical treatments [74,75,76] include alkaline, silane, acetylation, benzoylation, hydrogen peroxide, cisbutylated coupling agent, sodium chlorite, acrylation and acrylonitrile grafting, isocyanate, stearic acid, permanganate, triazine, oleoyl chloride, and fungicide treatments. An overview of the different chemical treatments of NPFs is schematically shown in Figure 7.

#### 3.2.1. Alkali Treatment

**Figure 8:**
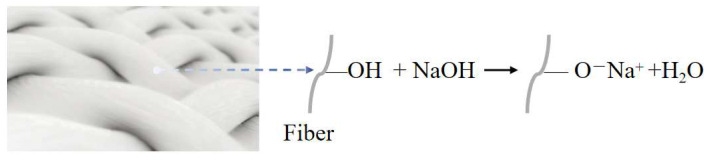
Reaction principle of the alkali treatment of NPFs.

Alkali treatment is a widely used chemical treatment of NPFs [79]. This method uses sodium hydroxide (NaOH) to modify NPFs, removing weak components such as lignin from the surface of the NPF, revealing cellulose molecules, increasing the number of reactive sites, and improving the surface roughness of the NPF, thus enhancing adhesion to the resin matrix [80]. Figure 8 shows the principle of the NaOH solution treatment of NPFs, and Table 2 describes the research on NaOH solution treatment of NPFs in recent years.

Alkali treatment can enhance interfacial performance but may weaken the tensile strength of NPF, as etching can cause pits on the fiber surface that may lead to stress concentration during load transfer. Therefore, when treating NPFs with alkali, attention should be paid to the pH value and treatment time. Some alkali treatments may also heat up, so the heating temperature needs to be controlled. The general temperature for alkali treatment is 40–60 °C.

#### 3.2.2. Silane Coupling Agent Treatment

**Figure 9:**
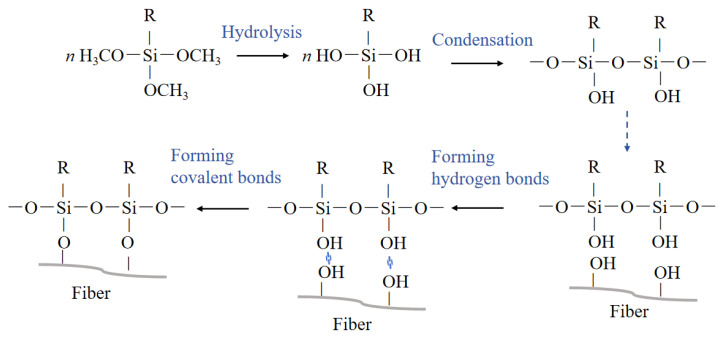
The main reaction process of a silane coupling agent in treating an NPF.

Silane coupling agent modification is an important chemical modification method. The main mechanism behind it is driven by the presence of bifunctional groups in the silane molecules, which can react with both the fibers and the resin matrix, establishing stable covalent connections between the NPF and resin [91]. Figure 9 shows the main reaction processes of treating an NPF with a silane coupling agent, including hydrolysis, condensation, formation of hydrogen bonds, and covalent bonds [92]. Table 3 introduces the research on the treatment of NPFs with silane coupling agents in recent years.

#### 3.2.3. Graft Copolymerization (GC)

**Figure 10:**
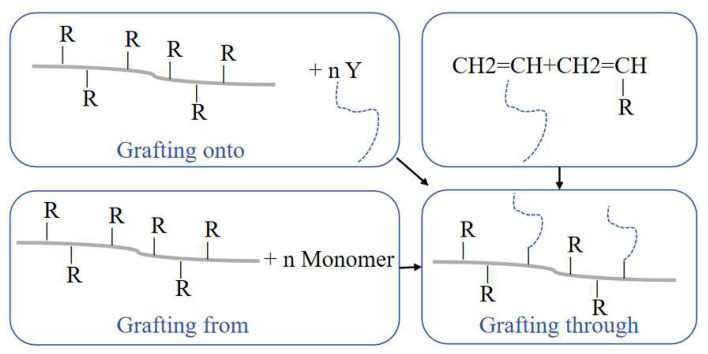
Graft copolymerization treatment of NPF.

**Figure 11:**
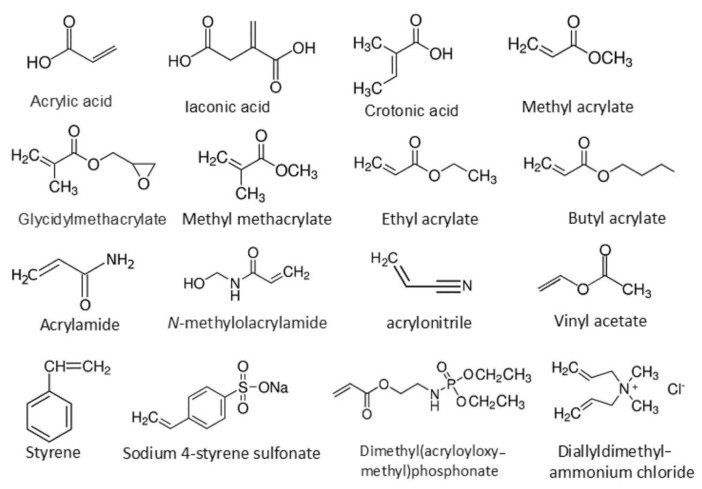
List of monomers commonly used for the modification of NPFs by graft copolymerization [102].

GC is a very effective modification method for the surfaces of NPFs. The reaction mechanism involves cellulose and monomer grafting without completely destroying the cellulose’s properties, ultimately resulting in the natural fiber’s molecular chain being grafted with copolymers of different properties. The monomers mainly include acrylic acid, acrylonitrile, and others [101]. Figure 10 is a schematic diagram of NPF graft copolymerization, where Y and R represent the grafting monomers and functional groups on the fibers, respectively. Figure 11 shows a list of common monomers used for the modification of NPFs through graft copolymerization. The grafting monomers need to be selected based on the type of fiber. Table 4 introduces the recent research on the treatment of NPFs with GC.

#### 3.2.4. Acetylation Treatment

**Figure 12:**
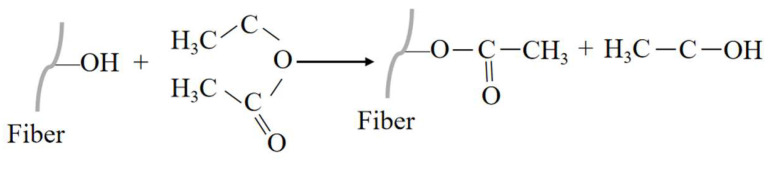
Reaction principle of the acetylation of an NPF.

Acetylation is the esterification of NPFs with acids, anhydrides, etc. Esterification is the process by which the hydroxyl group (-OH) of an NPF reacts with the acetyl group (CH3COO-) [110]. The reaction principle is shown in Figure 12. The polar hydroxyl groups in the fibers are replaced by acetyl groups, which reduces the polarity of the NPF and is more conducive to the dispersion of the NPF in the resin, thus improving the interfacial bonding properties between the fibers and the resin [111]. Table 5 introduces the research on the treatment of an NPF with acetylation in recent years.

#### 3.2.5. Benzoylation Treatment

**Figure 13:**
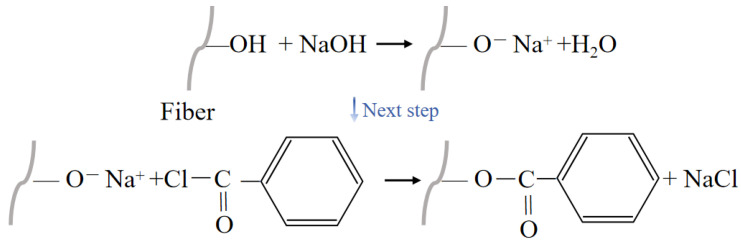
Benzoylated NPF.

Benzoyl chloride can react with the hydroxyl groups in an NPF (as shown in Figure 13) to form benzoyl esters, thereby increasing the fibers’ lipophilicity and improving their adhesion to non-polar resins. For example, Thulasiram [118] performed alkali, silane, permanganate, and benzoylated surface treatments on coconut tree secondary flower leaf stalk (CSF), and the results were found to show that the CSF benzoylated treatment significantly increased the tensile strength (650.54 MPa) and Young’s modulus value (1.89 GPa) in TGA. Additionally, the benzoylated CSF exhibited the highest retention temperature of thermal stability at 567 °C compared with other treatments. Sibakanta [119] chemically treated Bauhinia vahlii (BV) fibers with sodium hydroxide, sodium chlorite. The benzoylation treatment resulted in the highest tensile strength of the benzoylated BV fibers, which was 128.56 MPa, and a Young’s modulus of 8.34 GPa.

#### 3.2.6. Permanganate Treatment

**Figure 14:**
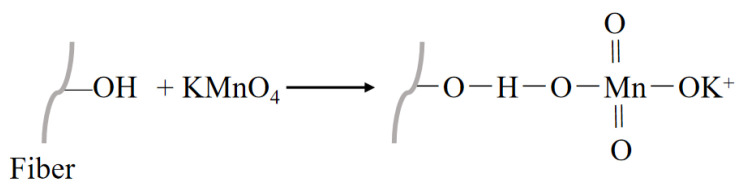
Treatment of an NPF with potassium permanganate.

Potassium permanganate (KMnO4) is a strong oxidant that oxidizes hydroxyl groups (-OH) on the fiber surface to form polar groups, such as carboxyl groups (-COOH), thereby increasing the chemical activity and hydrophilicity of the fiber surface [120]. However, the effectiveness of permanganate treatment depends on the surface properties of the plant fibers, as permanganate treatment may lead to fiber deterioration, limited compatibility with specific polymers and sensitivity to manufacturing processes [121]. Figure 14 illustrates the mechanism of treating fibers with potassium permanganate. Table 6 introduces recent research on the treatment of NPFs with potassium permanganate.

#### 3.2.7. Isocyanate Treatment

**Figure 15:**
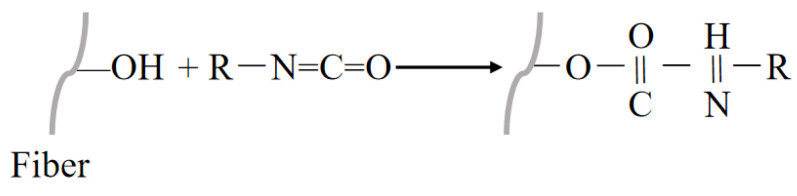
Treatment of an NPF with isocyanate.

The isocyanate element (N = C = O) in the isocyanate treatment (R-N = C = O, where R denotes a free radical) reacts with the hydroxyl group, leading to the formation of a carbamate bond and rendering the fiber hydrophobic [129]. The principle of the isocyanate treatment is shown in Figure 15. Isocyanate coupling agents usually contain a second functional group that forms a covalent bond with the substrate, considered to be strongly bonded. Manggar [130] used polymerized diphenylmethane diisocyanate impregnation of ramie fibers, and the experimental results were found to reveal that the modified fibers were more thermally stable, while the tensile strength of the composites with polyurethane was increased by 13%. Shi [131] used a class of nonionic cross-linking agents with isocyanate reactive groups for Lyocell, and the fiber was treated with PMDI at 20 g/L, 175 °C and 80 s. The fibrous chattering tendency was greatly reduced under the treatment conditions. In addition, good wrinkle resistance was added to the Lyocell fabric.

#### 3.2.8. Maleic Anhydride Treatment

Maleic anhydride, when employed as a modifier, is capable of esterifying the hydroxyl groups (-OH) present in cellulose fibers, thereby forming ester bonds [132]. This modification reaction results in an increase in the quantity of carboxyl groups (-COOH) present within the fiber. The introduction of the carboxyl group markedly enhances the hydrophilicity of the fiber, as it is capable of forming more robust hydrogen bonds with water molecules. This property is of critical importance in the manufacture of composites, as it enhances the bonding between the fibers and aqueous or polar resins, thereby improving the overall performance of the composite.

The increase in carboxyl groups on the surface of maleic anhydride-modified cellulose fibers enhances interaction with the resin matrix, contributing to the mechanical strength and durability of the composites. Consequently, the modification of maleic anhydride does not merely maintain the adhesive properties of cellulose fibers, but also markedly enhances their applicability in composites and reinforces their adhesive strength with the matrix [133]. Younesi-Kordkheili [134] investigated the effect of maleic anhydride-treated nano-lignin as a coupling agent on the various properties of wood fiber–polypropylene composites and their results were found to show that the glass transition temperature of the nano-lignin was reduced from 130 °C to 100 °C by esterification. Simultaneously, the nano-lignin content was increased from 1 wt% to 5 wt%. Gazi Md Arifuzzaman [135] treated selected okra bast fibers with bleaching agents, applied treatments of maleic anhydride, and grafted them with vinyl acetate. The tensile strength of the prepared polyvinyl alcohol-based composites ranged from 33.8 to 55.1 MPa, with a modulus of elasticity ranging from 1.8 to 2.6 GPa and an elongation at break varying from 2.8 to 10%. The chemical treatment resulted in enhanced mechanical properties, whereas the rise in fiber content led to a decline in tensile strength, stiffness, and elongation, as well as an increase in water absorption.

#### 3.2.9. Stearic Acid Treatment

**Figure 16:**
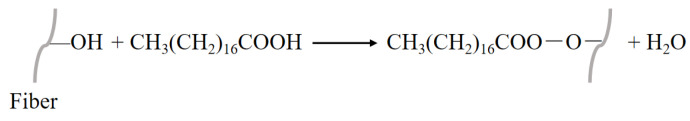
Treatment of an NPF with stearic acid.

Stearic acid forms a stearic acid layer on the fiber surface through a chemical reaction between its carboxyl group (-COOH) and the hydroxyl group (-OH) on the fiber surface to form an esterification reaction [136,137]. The reaction principle is shown in Figure 16. This modification reduces the hydrophilicity of the fiber and enhances its compatibility with the non-polar polymer matrix. Mohanta [138] investigated stearic acid treatment of bamboo fiber (BF) for the preparation of bamboo fiber high density polyethylene composites (BF/HDPE) and found that the tensile strength of stearic acid-treated BF/HDPE was increased by 9.26% and that of stearic acid-treated BF-rHDPE was increased by 16.5%. Claudia [139] used stearic acid and castor oil reagents as modifiers for short bagasse fibers and suggested that stearic acid treatment of cementitious short bagasse fiber composites with a fiber content of 2 wt% results in the optimum mechanical and physical properties.

#### 3.2.10. Peroxide Treatment

**Figure 17:**
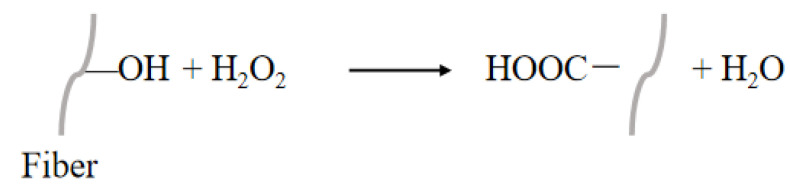
Treatment of an NPF with peroxide.

Peroxides, including hydrogen peroxide and dibenzyl peroxide (DCP), function as potent oxidants, capable of oxidizing organic compounds on the fiber surface. This oxidation process generates new functional groups on the fiber surface, such as carboxyl groups (-COOH) and ketone groups (>C=O), which significantly enhance the chemical bonding between the fiber and the resin matrix upon introduction. The reaction principle is shown in Figure 17. As a result of this chemical modification, the functional groups on the fiber surface form a greater number of chemical bonds with the resin matrix, thereby enhancing the interfacial adhesion between the two. This modification method is of great consequence for the enhancement of the mechanical properties and durability of the composites, as it facilitates the reduction of interfacial voids between the fibers and the matrix, thereby improving the efficiency of stress transfer and, consequently, the overall performance of the composites [140]. Chauhan [141] investigated the effect of the benzoyl peroxide (BP) treatment of Grewia optiva fibers on fiber diameter and found that the reduction in fiber diameter due to BP treatment was about 57%. Fu [142] used alkaline oxygenation for extracting reed straw fibers and proposed the optimum process parameters for alkaline oxygenation in one bath method, as follows: a dosage of sodium hydroxide of 35 g/L, a dosage of hydrogen peroxide of 30 mL/L, a treatment temperature of 85 °C, and a steaming time of 2 h. Under these conditions, the degumming rate was 54.30%.

#### 3.2.11. Triazine Treatment

The principle of the triazine (C3H3N3) treatment of NPFs mainly involves chemical modification by reacting triazine derivatives with hydroxyl groups on the surface of the NPF to form covalent bonds. This grafting can alter the surface properties of the fibers, such as by improving hydrophobicity, enhancing thermal stability, etc. Xu [143] investigated the propensity and mechanism of protofibrillation of Lyocell fibers treated with sodium 4,6-dichloro-2-dioxo-1,3,5-triazine, and the results were found to show that the chemical reaction takes place on the surface of crystalline or amorphous zones of cellulose. The chemical reaction process does not change the structure of Lyocell fibers and has little effect on the mechanical properties and dyeing ability of Lyocell fibers.

### 3.3. Other Treatments

#### 3.3.1. Fungal Treatment

Fungal treatment represents a biological treatment method. The fundamental premise of NPF remediation hinges on a biodegradation process wherein particular fungal strains secrete a suite of enzymes, including laccase, manganese peroxidase, and lignin peroxidase, which are adept at breaking down lignin. Similarly, cellulases act on cellulose and hemicellulose [140]. The collective action of these enzymes results in the partial hydrolysis of lignin and hemicellulose within the fiber, thereby exposing a greater quantity of cellulose microfilaments and increasing the surface roughness and accessible area of the fiber. Following the application of enzymes, alterations to the fiber surface are observed, which may enhance its ability to adhere to non-polar resin matrices. This is due to the fact that the enzyme treatment may remove certain impediments to adhesion, such as the presence of hemicellulose and lignin on the cellulose microfilaments. This results in an increase in the irregularity of the fiber surface and the number of active sites [62].

#### 3.3.2. Nanotechnology Treatment

**Figure 18:**
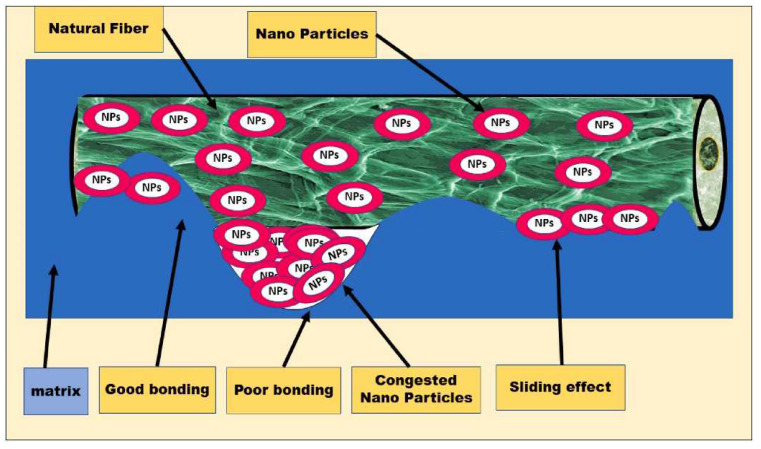
Integration of nanoparticles into an NPF [62].

Nanotechnology can chemically or physically modify the surface properties of an NPF, for example, through techniques such as grafting, coating or plasma treatment, to increase the hydrophilicity or hydrophobicity of the fiber surface and improve its compatibility with the resin matrix [144]. Figure 18 illustrates the principle of the nanotechnology treatment of NPFs. Godias Tumusiime [145] treated the surface of silk fibers with ZnO nanoparticles, enhancing the surface adhesion of the fibers to the polyester resin, thus improving the mechanical properties of the resulting composites. Additionally, nanotechnology can optimize the microstructure of the composites by reducing the aggregation of fibers and increasing their dispersion in the matrix, thus improving the homogeneity and properties of the materials. Additionally, nanotechnology can confer new functions when introduced to NPFs, such as by imparting antibacterial, flame-retardant, and UV-resistant properties. For example, the synthesis of nanoparticles on Agave tequilana Weber var. azul fibers (ATF) endowed it with antimicrobial activity against Streptococcus enterica [146]. Forkan coated graphene material on jute fibers and increased the shear strength of the jute–epoxy interface by 236% and the tensile strength by 96% when compared with untreated fibers [147]. Amer added graphene to flax fibers and experimentally found that the hygroscopicity resistance of the flax fibers was significantly improved with the addition of 0.5% graphene [148]. Wang grafted flax fiber sheets with multi-walled carbon nanotubes, silane coupling agent and TiO_2_ nanoparticles. Their results were found to show that the FRP composites grafted with multi-walled carbon nanotubes had higher tensile and interlaminar shear strengths than those grafted with the same nano-TiO_2_ particles [149]. Li [150] successfully coated carboxyl-functionalized carbon nanotubes (COOH-CNT) on flax fibers using a ‘dip or spray-dry’ process. Their results were found to show that the maximum enhancements of interfacial shear strength, type I interlaminar fracture toughness and interlaminar shear strength of flax fiber-reinforced epoxy composites were 26%, 31% and 20%, respectively.

Overall, the application of nanotechnology in natural fiber treatment not only promotes the development of new materials, but also provides new ideas and methods for the upgrading of traditional materials. With in-depth research and technological progress, nanotechnology will show its unique value and potential in more fields in the future.

### 3.4. Future Development of Treatments

**Figure 19:**
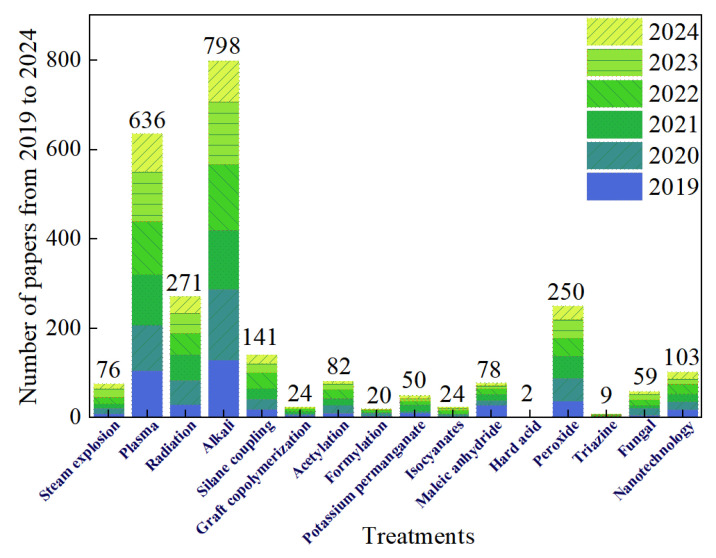
Research papers on NPF treatment methods 2019–2024.

There are many methods for treating NPFs, and different methods can have varying effects on the functionality of the NPF. Therefore, the treatment process steps can be tailored to the desired functionality of the NPF and various treatment processes can be combined to synergistically enhance the material properties of the NPF. Figure 19 summarizes the number of research papers on different treatment methods from 2019 to the present day, clearly showing that alkali treatment has the highest number. This indicates, on one hand, the widespread application of alkali treatment, and on the other hand, its effectiveness in enhancing the material properties of an NPF. It is worth noting that many studies have used hybrid methods, in which alkali treatment is often used as a pretreatment method, mainly because the operation difficulty and cost of alkali treatment are relatively low, and the raw materials are easily obtained [151]. The second most researched method is plasma treatment. As a physical treatment, plasma offers high processing efficiency, can handle large quantities, and does not produce chemical pollution. Its significant enhancement effect on the material properties of NPFs has made it very popular, and this trend is expected to continue in the future.

In Table 7, the advantages and disadvantages of the various treatment methods are compared and the characteristics of various treatment methods are evaluated. The evaluation criteria consider the cost of the equipment and materials used in the treatment process, the treatment time, the difficulty of operation, sustainability, whether toxic substances are used or produced, the durability of the treated material and whether high temperatures are required. Physical methods are primarily costly in terms of equipment but show significant advantages in all other aspects, whereas chemical treatment methods mainly result in chemical pollution and are not environmentally friendly. It is worth noting that the biological treatment method, specifically fungal treatment, has not been widely applied currently, primarily due to its long treatment time and low efficiency. In the future, perhaps more efficient fungi can be used, or a mixture of biological and other methods can be used to improve the treatment efficiency. In recent years, the newly emerging technology of nanoprocessing has shown great potential for application, a potential limited mainly by the cost of the technology and by the need to improve the technology of extracting cellulose. When these technological means and equipment mature in the future, perhaps nanotechnology processing methods can become an efficient means by which to enhance the performance of NPF materials.

## 4. Development of Thermoplastic NPFRPs

The performance of NPFRPs depends greatly on the choice of matrix material. Not only does the matrix act as a protective layer for the fibers against harsh environments and wear and tear, it also significantly enhances the overall durability of the material and improves surface texture. Currently, the most commonly used substrates in NPFRPs are synthetic polymers derived from petroleum-based sources, which are widely used for their light weight and low processing temperatures.

**Figure 20:**
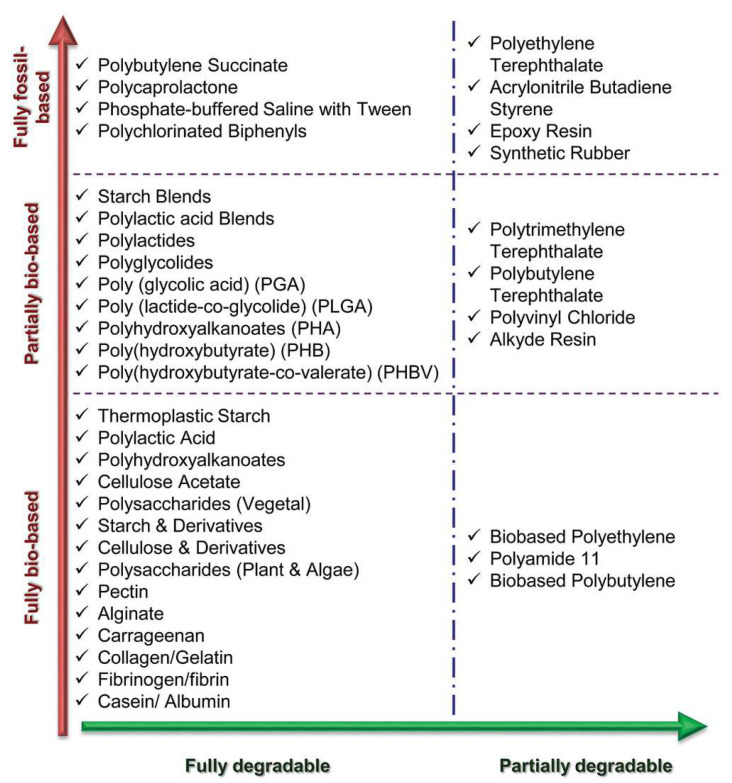
Types of polymeric matrices [155].

However, typically NPFs exhibit thermal instability at temperatures above 200 °C. In view of this, only those thermoplastics and thermosets that can soften below 200 °C are suitable as substrates for NPFRPs [152]. The commonly used polymer matrices in NPFRPs are carefully categorized according to their biodegradability, as shown in Figure 20, which helps in the selection of matrix materials that are environmentally friendly and suitable for processing and application needs.

Currently, most thermoplastic and thermoset matrix materials are derived from petrochemical sources, i.e., synthesized through petrochemical processes. However, with the development of bio-based materials technology, some thermoplastics, such as polyamide 46 (PA46) and bio-based polypropylene (Bio-PP), as well as thermosets such as Ecoflex (a biodegradable polyester), have been successfully developed from renewable bio-based sources. Among thermoplastic matrix materials, polyethylene (PE), polypropylene (PP), polyvinyl chloride (PVC) and polystyrene (PS) are common choices. These materials are fluid when heated, can be molded by conventional plastics processing techniques such as injection molding and extrusion, and can be reshaped when heated without any change in chemical structure. On the other hand, thermosetting matrices such as epoxy resins, phenolic resins and unsaturated polyesters form a three-dimensional network structure during the curing process, providing excellent thermal stability, chemical resistance and creep resistance.

Table 8 presents the characteristics of different thermosetting resins. Epoxy resins, unsaturated polyester resins, and phenolic resins—three widely used thermosetting polymers—play significant roles in the fields of electronics, electrical applications, and structural composite materials. However, these materials face challenges in recycling and contribute to a higher carbon footprint throughout their lifecycle, potentially having adverse effects on the environment. The environmental burden is further increased when synthetic fibers are used as reinforcing agents in composite materials. In contrast, NPFs offer an environmentally friendly alternative, being not only renewable and biodegradable but also characterized by lower energy consumption and carbon footprint in their production processes. Although NPFs may not match synthetic fibers in terms of their thermo-physical properties, their performance can be significantly enhanced through appropriate treatment and modification, rendering them as viable eco-friendly options for numerous applications. Therefore, by leveraging the green advantages of NPF, we can effectively reduce the carbon footprint of composite materials and propel the composite materials industry towards a more sustainable direction.

Bioresins, which are polymers derived from renewable plant resources and synthesized through the polymerization of plant sugars and oils, have emerged as a sustainable alternative to traditional petroleum-based polymer resins. Based on their biobased composition and biodegradable properties, bioresins can be classified into three main types: fully biobased and biodegradable, partially biobased and biodegradable, and partially biobased non-biodegradable [153,154]. Examples of these resins include starch, polylactic acid (PLA), polyhydroxyalkanoates (PHA), bio-based phenolic resins, hydroxymethylfurfural (HMF), alginate, dextran, xanthan gum, and cellulose. Among these bioresins, polylactic acid (PLA) is the preferred material due to its excellent processing properties and biodegradability [155]. Despite the significant cost-effectiveness advantages of bioresins and their environmentally friendly properties, such as degradability, reduced greenhouse gas emissions, non-toxicity, and energy-efficient production processes, bioresins’ thermal stability and mechanical properties are still deficient when compared with traditional petroleum-based resins, which limits their widespread use in high-performance applications. To address this issue, researchers are working to improve the overall performance of bioresins through chemical modification, blending techniques, and the use of enhanced fillers, with the aim of achieving wider adoption in industrial applications.

The vast majority of thermoplastic resins are of a class of polymeric materials that can be plasticized when heated and hardened when cooled. These are used in a wide range of industrial applications and have significant advantages. Thermoplastic resins can be molded by a variety of processing methods such as injection molding, extrusion, and blow molding, which are highly productive and easy to automate [156]. Additionally, thermoplastic resins can be re-melted and re-shaped when heated, which means they can be recycled and reused, reducing material waste. Compared with thermoset resins, thermoplastic resins are typically lower in cost and use relatively less energy during processing. Thermoplastic resins can provide excellent mechanical properties such as high strength, high toughness and good wear resistance. Furthermore, many thermoplastic resins are highly resistant to chemicals and are suitable for use in a variety of chemical environments. In addition, thermoplastic resins can be manufactured in a variety of colors and clarity by adding pigments or using transparent resins.

Thermoplastic resins typically require heating and melting at high temperatures during preparation and molding, increasing the cost of equipment in the preparation process. Additionally, the molding temperature for thermoplastic resins is often higher than the service temperature of the NPF, potentially decreasing the thermal stability of the NPF during processing and significantly limiting the application of thermoplastic resins in NPFRPs. However, it is worth noting that, in 2015, the French company Arkema released a liquid thermoplastic resin that can be cured at room temperature—Elium resin [157]. Unlike other thermoplastic resins that need to be preprepared as a semi-finished product and then heated and melted to be molded, Elium resin is in a liquid state at room temperature, and can be molded using vacuum-assisted resin transfer molding (VARTM) under the effect of an initiator. Elium resin is liquid at room temperature and can be directly polymerized in-situ at low temperature under the action of an initiator using VARTM, vacuum-assisted resin injection (VARI) or pultrusion, which is a simple manufacturing process with low energy consumption. Elium resins can be cured at room temperature, which greatly reduces the cost of energy consumption in production. To date, over 1000 research papers have been published on Elium resins, and numerous studies have demonstrated their excellent mechanical properties, indicating their potential to become superior alternatives to conventional epoxy resins [158]. However, the majority of current research on Elium-FRPs focuses on synthetic fibers as the reinforcing phase, with relatively little attention given to the application of NPFs. In a recent study by Shen et al. [159], the tensile and flexural mechanical properties of carbon fiber/Elium resin were analyzed under different preparation process parameters and compared with those of conventional carbon fiber/epoxy resin (CF/EP) composites. The results were found to show that the carbon fiber/Elium resin composites (40 °C) series with a heating time of 90–180 min performed the best in terms of tensile and flexural strengths, which were improved by 4.44–15.07% and 28.94–43.98%, respectively, compared with CF/EP. Kazemi [160] investigated the resistance to the low-tensile and bending mechanical properties of ultra-high molecular weight polyethylene fiber (UHMWPE)/Elium resin composites for low-velocity impact resistance and compared them with UHMWPE/epoxy composites. The results were found to show that the newly developed room-temperature thermoplastic structures could achieve higher impact loads and as low as 40% of the absorbed energy as compared with the thermoset structures. In addition, replacing the thermoset resin with a thermoplastic resin was found to significantly improve the structural integrity of the laminate by 240%. Elium resin-based FRPs (Elium-FRPs) not only exhibit excellent mechanical properties, but also have similar flexural strength reduction and damage modes as epoxy resin under dry, wet and diesel conditions [161].

Nowadays, reports on Elium–NPFRPs are mainly focused on the characterization of the tensile, flexural, compressive, shear and fatigue properties of Elium resin matrix with flax fiber reinforcement. Chilali [162] et al. analyzed the static tensile and in-plane shear properties of twill flax fiber-reinforced Elium. Haggui [163,164] et al. evaluated the fatigue bending properties, tensile failure and damage behavior of bi-directional flax fiber-reinforced Elium resin matrix composite laminates under static and cyclic loading, and noted that flax/Elium composites exhibit good fatigue resistance. Baley [165] et al. comparatively investigated the tensile and compressive properties of flax fiber-reinforced Elium resin matrix composites and noted that the interfacial bond strength between the flax fiber and Elium resin was weak. However, there is no report on the study of the real mechanical property changes during service, such as impact resistance or UV aging performance.

In recent years, the zero waste blade research (ZEBRA) consortium has utilized Arkema’s Elium resin and a new high-performance glass fiber fabric to create the first 100% recyclable prototype wind turbine blade. Elium-based composite parts are recycled using advanced chemical recycling methods, and recycling rates can be as high as 100%, with little or no reduction in resin properties [166]. Therefore, the recycling of Elium–NPFRP composites is no longer a problem, and Elium–NPFRP composites can be a very useful option in the future development of green and sustainable composites.

Finally, Table 9 compares the advantages and disadvantages of different recyclable resins. Compared with other resin systems, Elium resin has the characteristic of room temperature curing, which can reduce the cost of NPFRP preparation equipment. Although current epoxy resins can also be dissolved and recovered through supercritical and subcritical fluids, their toughness is poor, and they are prone to produce subtle cracks when subjected to low-speed impact, which can break the continuity of composite materials and have adverse effects on their mechanical properties. And Elium resin has good fracture toughness, which increases the application scenarios of Elium–NPFRP. Elium can achieve a recovery rate exceeding 90% through acetone recovery, and the acetone can also be recycled. Compared with other resin recovery processes listed in Table 9, the carbon footprint of the Elium recovery process is relatively low. Thus, throughout the entire lifecycle of Elium–NPFRP, the carbon footprint of the preparation and recovery process is lower compared with other thermosetting resins.

## 5. Conclusions and Outlook

According to the current market trends, NPFRPs are experiencing extensive growth, with promising applications in the automotive, ballistic protection, biomedical, food packaging and construction industries. There are many types of NPF, and their mechanical and chemical properties can vary greatly, so the choice of fiber type needs to be based on a combination of fiber properties and application scenarios. For future complex application scenarios and functional requirements in the future, blended NPFs can be used for functional design to achieve target performance requirements. Meanwhile, surface treatment of fibers is a common method to further enhance the material properties of NPFs. Through in-depth research, it has been found that there are numerous studies at home and abroad on the enhancement of the material properties of NPFRPs by the surface treatment of NPFs, and good results have been achieved by physical, chemical and biological treatments, etc. However, all of these methods have some shortcomings and there is much room for improvement. Regarding future enhancement of the treatment methods, there is a great prospect for the development of research into, and application of, nanotechnology and hybrid treatment methods.

Finally, NPFRPs based on thermoplastic resins can substantially highlight the green and sustainable advantages of NPFs. This paper has also briefly summarized the advantages and disadvantages of traditional thermoset and thermoplastic resins and proposes design ideas associated with Elium–NPFRP materials, which are expected to further promote the application of NPFRPs in the field.

## Figures and Tables

**Table 2:** Alkali treatment of NPFs.

NPF	Treatment Conditions	Remarks	Ref.
Bamboo	Soak in 2% NaOH solution for 1 h.	The surface polarity of bamboo fibers decreases, reducing agglomeration and increasing the dispersion rate of bamboo fibers in the mixture by 9%.	[81]
*Pulicaria gnaphalodes*	Soak in 5% NaOH solution for 90 min.	The crystallinity and thermal stability of fibers is significantly improved.	[82]
Hemp	Soak in 3% NaOH solution for 1 h.	The tensile strength, flexural strength, and impact strength of the hemp fiber/vinyl ester composite material is improved after alkali treatment.	[83]
*Portulaca quadrifida*	Soak in respective alkaline solutions of 5%, 10%, and 15% for 1 h.	The tensile strength of fibers treated with 15% alkali was found to be 5.45 MPa, while that of untreated fibers was 2.70 MPa.	[84]
*Hibiscus canescens stem*	Soak in 5% NaOH solution for 1 h.	The Tg increases by 6%, the cellulose content increases by 9.03%, and the fiber density increases by 2.06%.	[85]
*Sambucus ebulus*	Soak in 5% NaOH solution for 30 min.	The fiber’s elongation at break increases by 0.5%, and the Tg temperature increases by 17%.	[86]
Coconut petiole	Soak in 10% alkaline solution for 2 h.	The composite material composed of polylactic acid resin shows a 150% increase in impact strength and a 50% increase in flexural modulus after treatment.	[87]
*Lankaran acacia*	Soak in 20% alkaline solution.	The composite material composed of epoxy resin shows an 8.52% increase in tensile strength, a 15.50% increase in bending strength, and a 42.6% increase in impact strength after treatment.	[88]
*Himalayacalamus falconeri*	Soak in 5% alkaline solution for 1 h.	The cellulose content increases by 6%, fiber density increases by 4%, and fiber tensile strength increases by 49%.	[89]
*Mucuna atropurpurea cellulose*	Soak in 5% NaOH solution for periods of 15, 30, 45, 60, and 75 min.	Tensile strength increases from 274.6 ± 29.5 to 307.3 ± 24.12 MPa and tensile modulus increases from 2.88 ± 1.026 to 4.633 ± 0.94 GPa.	[90]

**Table 3:** Silane coupling agent treatments of NPFs.

NPF	Treatment Conditions	Remarks	Ref.
Waste corrugated paper	Three amino silane coupling agents, KH550, KH560, or KH570, at treated concentrations of 2 wt%, 4 wt%, 6 wt%, or 8 wt%, treatment time of 3 h.	The Tg increases.	[93]
Nettle	Two different silane coupling agents, TES-PM and APTES, at concentrations of 0.5 wt%, 1.0 wt%, 2.5 wt%, and 5.0 wt% and a treatment time of 1 h.	The static mechanical properties (tensile, bending, and impact), dynamic mechanical properties (storage and loss modulus and damping factor), and biodegradability of the nettle/polylactic acid composite materials are improved.	[94]
Ramie	Amino silane coupling agent KH550 at a concentration of 1 wt% and with a treatment time of 2 h.	The tensile and bending properties of the composite materials based on block copolymers are significantly improved.	[95]
Flax/kenaf	Concentration: 2 wt%, time: 2 h and 4 h.	The increase in fiber density results in a 6% increase in fiber tensile strength.	[96]
Lignocellulose	Trimethoxysilane (GLYMO) at concentrations of 3, 5, 7, and 10 wt% and a treatment time of 30 min.	Composite materials based on polylactic acid improve tensile strength and modulus by 14% and 27%, respectively.	[97]
Coconut	Three types of silane agents: GLYMO, vinyltrimethoxysilane (VTMS), and tetraethoxysilane (TEOS).	The thermal stability of fibers is improved.	[98]
Henequen	Silane agents: maleic anhydride (MA) and diisopropylbenzene peroxide, concentration of 5 wt%.	The tensile strength and modulus of the fiber increased by 68% and 32%, respectively, and the thermal stability increased by 12 °C.	[99]
Linen/cotton	3-aminopropyltriethoxysilane (3-APS) at a concentration of 2 wt% and treatment time of 3 h.	The tensile strength and impact strength of composite materials based on epoxy resin have been improved.	[100]

**Table 4:** Graft copolymerization treatment of NPFs.

NPF	Treatment Conditions	Remarks	Ref.
Cellulose diacetate (CDA)	L-lactide (LA)	When the mass ratio of L-LA/CDA is 4:1, the grafting rate is highest.	[103]
*Miscanthusxgiganteus stem fragments* and short flax	Dimethyl (methacryloyloxy) methylphosphonate (MAPC1)	The storage temperature, reaction temperature, and concentration of Fe^2+^ affected the grafting yield.	[104]
Bagasse cellulose (BC)	Dual functional glycidyl methacrylate (GMA)	The grafting degree of BC-GMA reached 27.02%, and the highest thermal decomposition temperature was 359.31 °C.	[105]
*Yucca filamentosa*	Ethyl methacrylate	The acid resistance of the fiber increases, and the moisture absorption rate decreases.	[106]
*Luffa cylindrica*	3-(trimethoxysilyl) propyl methacrylate	The removal rates of Pb^2+^, Ag, Cu^2+^, Cd^2+^, Co^2+^, and Ni^2+^ were 97.50%, 95.50%, 75.50%, 66.50%, 59.00%, and 51.00%, respectively.	[107]
Wheat straw (WS)	Methyl methacrylate (PMMA)	PMMA grafting onto WS greatly improves its adhesion to oil.	[108]
*Cannabis indica*	Benzoyl chloride and acrylic acid/acrylonitrile	The moisture absorption and chemical resistance of fibers increases.	[109]

**Table 5:** Acetylation treatment of NPFs.

NPF	Treatment Conditions	Remarks	Ref.
Henequen	Treated in a solution of acetic anhydride at 60 °C for 1 h.	The lignin content of the fibers decreased by 20%, and the water absorption capacity was reduced.	[112]
Windmill palm	N-dimethylformamide (DMF) and acetyl chloride, with a treatment time of 15 min.	The hydrophobicity of the fibers was improved and the hydrostatic contact angle was increased to over 145°. The acoustic performance of the fiber mats was also improved, with an average sound absorption coefficient of 0.47.	[113]
Date-plam	Treatment in Soxhlet acidified sodium chloride solution (30 wt% acetic acid, 5% sodium chloride) at 105 °C for 2 h.	Increased ultimate tensile strength and modulus of elasticity of the fibers, and increased the crystallinity of the fibers by 32%.	[114]
Sisal hemp	For the alkali acetylation, the alkali-treated sisal was initially immersed in acetic acid for 1 h and then transferred to a solution containing 250 mL of acetic anhydride and 0.1 wt% of H_2_SO_4_ as a catalyst for 5 min.	In composites with polyethylene terephthalate (PET), the tensile modulus of the composite was increased by 137%, the flexural strength was significantly increased by 66.6–190%, and the flexural modulus was increased by 110.5–410.0%.	[115]
Wheat straw	A mixture of acetic anhydride and sulphuric acid, solution temperature of 20–100 °C and a treatment time of 1–12 h.	The complex modulus of asphalt dispersions formulated with acetylated samples was four times higher than that of pure asphalt, and the phase angle indicated an improvement in elastic behaviour parameters.	[116]
Luffa fiber	Treated in a 1:1 mixture of acetic acid and acetic anhydride solution for 1 h.	Tensile and flexural strength increased by 4.3 per cent and 0.4 per cent, respectively.	[117]

**Table 6:** Potassium permanganate treatment of NPFs.

NPF	Treatment Conditions	Remarks	Ref.
Sisal hemp	The fibers were treated with different concentrations (0.03%, 0.06%, 0.09%) of KMnO solution in a water bath heated at 40 °C for 15 min.	The tensile load of sisal fibers treated with KMnO increased from 26 N to 98 ± 1.8 N.	[122]
*Calotropis Gigantea Bast*	The alkaline pretreated fibers were first treated by immersion in potassium permanganate solution for 1 min.	The tensile strength was increased by 21.54% and the Tg temperature was increased by 20 °C from 230 to 250 °C.	[123]
*Furcraea Foetida*	Treatment in a solution of 0.5 wt.% KMnO_4_ for 30 min.	There was a 16% increase in fiber tensile strength and 8% increase in IFSS.	[124]
Butea Parviflora	Treated first in 0.1 wt% NaOH solution for 20 min, then immersed in 0.1 wt% potassium permanganate for 15 min at 27 °C.	Higher tensile strength (198 MPa) and Young’s modulus (4.40 GPa) of treated fibers compared with untreated fibers (untreated: tensile strength—92 MPa, tensile modulus—2.16 GPa).	[125]
Banana	Pre-treated with 0.06% NaOH and then treated with 0.003% KMnO4 solution for 3 min.	There was a 65.92% increase in tensile strength.	[126]
Bamboo	The fibers were treated by immersion in different concentrations of potassium permanganate solution (0.25%, 0.5%, 0.75% and 1%) for 3 min at room temperature.	There was a 25% increase in tensile strength, 14.08% increase in cellulose content, and 20% increase in crystallinity.	[127]
Zea mays root	Pretreatment was first carried out with 0.1 wt% sodium hydroxide solution for 10 min and then in a 0.1 wt% potassium permanganate solution for 10 min.	The water absorption increased, the crystallinity decreased (from 71.93% to 57.53%) and the maximum temperature limit decreased (525 °C to 511 °C).	[128]

**Table 7:** Characterization of different treatments.

Treatments	Cost	Efficiency	Difficulty of Operation	Green	Poisonous	Durability
Steam explosion	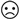	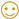	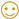	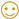	-	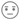
Plasma	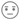	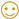	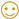	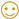	-	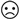
Radiation	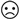	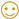	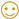	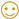	-	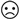
Alkali	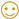	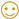	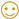	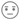	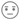	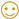
Silane coupling	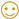	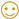	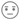	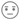	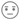	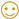
Graft copolymerization	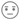	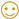	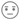	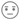	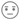	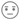
Acetylation	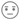	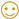	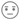	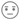	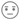	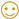
Formylation	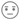	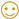	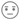	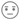	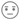	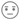
Potassium permanganate	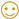	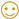	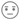	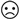	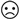	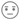
Isocyanates	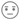	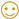	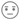	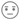	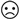	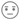
Maleic anhydride	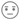	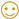	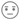	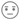	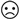	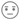
Hard acid	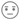	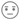	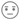	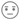	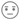	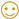
Peroxide	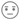	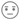	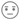	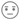	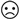	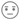
Triazine	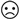	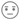	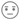	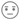	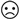	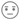
Fungal	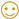	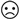	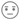	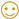	-	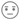
Nanotechnology	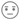	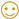	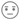	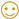	-	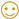

Note: The three expressions “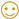 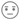 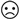
” represent high, general, and low respectively.

**Table 8:** The characteristics of different thermosetting resins.

Characteristic	Epoxy Resin	Unsaturated Polyester Resin	Phenolic Resin
Environmental advantages	Difficult to recycle, difficult to decompose after solidification.		
Thermal conductivity	0.2–2.2 W/(m·K).	0.25 W/(m·K).	0.1–0.3 W/(m·K).
Cure temperature	Room temperature–150 °C.	50–150 °C.	20–160 °C.
Thermal stability	Epoxy resin can generally withstand heat up to around 100 °C, and resins with special heat resistance levels can withstand heat up to 200 °C or above.	The thermal deformation temperature of most unsaturated polyester resins is 50–60 °C, while some resins with good heat resistance can reach up to 120 °C.	Phenolic resins are able to maintain high stability up to 200 °C.
Mechanical properties	Strong cohesive force, its mechanical strength is better than normal resin, and it can withstand long-term environmental stress.	High mechanical properties, including tensile, flexural and compressive strengths.	It has high compressive, tensile and flexural properties after curing, and is suitable for occasions where it is necessary to bear certain loads.
Cost	Easy to operate and low cost.	Low, and suitable for mass production.	
Biodegradability	Non-degradable after curing.		

**Table 9:** Advantages and disadvantages of different recyclable resins [167,168,169,170,171,172,173,174].

Resin	Advantages	Disadvantages	Remarks
Acrylonitrile butadiene styrene (ABS)	Has high impact strength, light weight, and chemical resistance properties. Various recycling technologies, such as mechanical and chemical, can be used.	The equipment and temperature requirements for the molding process are high.	Recycled ABS maintains over 90% of its original mechanical properties.
Polyethylene terephthalate (PET)	Superior transparency, durability, and recyclability. There are four main methods for PET recycling: primary recycling, mechanical recycling, enzymatic recycling, and chemical recycling.	Poor toughness, poor impact resistance, and a certain degree of brittleness. Long molding cycle, long molding cycle, high molding shrinkage rate, poor dimensional stability.	Microwave irradiation is an effective energy source for the depolymerization reaction of PET waste, with a short reaction time (6–7 min), which can significantly save energy.
Poly(vinyl chloride) (PVC)	Long service life, chemical resistance, and good mechanical properties.	PVC may produce harmful substances such as dioxins, vinyl chloride monomers, etc. during production and use.	The most recommended PVC recycling method is mechanical recycling. In some cases, the recycling of PVC materials can save up to 90% of energy compared with the energy input required for using raw materials.
Polyolefin (PO)	Good comprehensive performance in physics, chemistry, etc., and low price.	PO resins consist of weak Van Der Waals forces which result in their low melting and crystallization temperatures.	At 140 °C, the original POs were recovered using the n-hexane xylene/n-hexane dissolution/re precipitation technique without significant changes in the chemical structure of the POs.
Polyolefins (PEs and PP)	There are various processing methods, including extrusion, injection molding, blow molding, etc. Has good toughness and impact resistance.	Easy to age, especially under the influence of ultraviolet radiation, air oxidation corrosion may occur.	The mechanical properties of these two materials decrease with the increase of polymer cycling (reprocessing).
Epoxy	Low processing cost, easy operation, and good mechanical properties.	Poor resilience.	The use of supercritical and subcritical fluids for dissolution is a typical method for recycling waste epoxy resin composite materials.
Acrylic (Acrodur)	Has good mechanical properties, short cross-linking time, easy to handle and clean.	Acrodur is sensitive to moisture.	
Resin of methyl methacrylate (Elium)	Room temperature curing molding, good toughness.	Pungent odour.	Dissolvable and recoverable in acetone.

## Data Availability

No new data were created or analyzed in this study.
